# Aerobic exercise and its impact on musculoskeletal pain in older adults: a 14 year prospective, longitudinal study

**DOI:** 10.1186/ar1825

**Published:** 2005-09-19

**Authors:** Bonnie Bruce, James F Fries, Deborah P Lubeck

**Affiliations:** 1 Stanford University, Department of Immunology/Rheumatology, Palo Alto, CA 94304, USA; 2 Health Economics, Genentech/MS 241A, South San Francisco, CA 94080, USA

## Abstract

We studied the long term impact of running and other aerobic exercise on musculoskeletal pain in a cohort of healthy aging male and female seniors who had been followed for 14 years. We conducted a prospective, longitudinal study in 866 Runners' Association members (n = 492) and community controls (n = 374). Subjects were also categorized as Ever-Runners (n = 565) and Never-Runners (n = 301) to include runners who had stopped running. Pain was the primary outcome measure and was assessed in annual surveys on a double-anchored visual analogue scale (0 to 100; 0 = no pain). Baseline differences between Runners' Association members and community controls and between Ever-Runners versus Never-Runners were compared using chi-square and t-tests. Statistical adjustments for age, body mass index (BMI), gender, health behaviors, history of arthritis and comorbid conditions were performed using generalized estimating equations. Runner's Association members were younger (62 versus 65 years, p < 0.05), had a lower BMI (22.9 versus 24.2, p < 0.05), and less arthritis (35% versus 41%, p > 0.05) than community controls. Runners' Association members averaged far more exercise minutes per week (314 versus 123, p < 0.05) and miles run per week (26 versus 2, p < 0.05) and tended to report more fractures (53% versus 47%, p > 0.05) than controls. Ever-Runners were younger (62 versus 66 years, p < 0.05), had lower BMI (23.0 versus 24.3, p < 0.05), and less arthritis (35% versus 43%, p < 0.05) than Never-Runners. Ever-Runners averaged more exercise minutes per week (291 versus 120, p < 0.05) and miles run per week (23 versus 1, p < 0.05) and reported a few more fractures (52% versus 48%, p > 0.05) than Never-Runners. Exercise was associated with significantly lower pain scores over time in the Runners' Association group after adjusting for gender, baseline BMI, and study attrition (p < 0.01). Similar differences were observed for Ever-Runners versus Never-Runners. Consistent exercise patterns over the long term in physically active seniors are associated with about 25% less musculoskeletal pain than reported by more sedentary controls, either by calendar year or by cumulative area-under-the-curve pain over average ages of 62 to 76 years.

## Introduction

The prevalence of older adults in the United States is growing at a substantial rate. By 2030, nearly one-fifth of Americans will be in their sixties or older [1], which will have a considerable impact on public health. Numerous epidemiological and clinical studies have established that older adults who participate in regular physical activity are healthier and have a better quality of life than those who are inactive [2-4]. Regular exercise has also been shown to reduce pain in patients with knee osteoarthritis [5,6] and to help prevent mechanical low back pain [7]. In contrast, inactivity has been associated with greater pain with injury and has been associated with lower bone density and muscle tone [8]. On the other hand, some aerobic activities, such as running, have been found to result in increased risk for stress or other fractures [9,10]. Recurring trauma to soft tissue resulting from excessive physical activity conceivably could increase pain and disability [11]. Few studies have addressed the relationship between aerobic exercise and the perception of pain with advancing age.

To study the effect of exercise on disability and pain, our group [10] had investigated the relationship of running and its impact on musculoskeletal pain and disability in cohorts of Runners' Association members and community controls and Ever-Runners and Never-Runners who were followed prospectively for six years. In that study, no increase in joint pain or stiffness with age was observed in subjects who exercised often and intensely compared with their more sedentary counterparts. Pain was reduced, however, at all time points by about 25% in the exercising group. In fact, there was a slight decrease in pain for women who exercised over time.

In this investigation, we have extended that research in those cohorts. We have evaluated the association of vigorous physical activity with pain with advancing age after 14 years of follow-up. We hypothesized that those who regularly participated in running or other aerobic activity would report less musculoskeletal pain rather than more over the long term than did their inactive counterparts.

## Materials and methods

### Subjects

Sample selection and data collection methodology have been detailed previously [10]. Subjects were drawn from two groups: the Fifty-Plus Runners' Association with members across the United States and a Stanford University community-based random sample from the Lipid Research Clinics Study (community controls) which provided access to a sample that was similar in age to the Runners' Association. In this analysis, all subjects with at least two annual questionnaires were included. A total of 961 men and women (538 Runners' Association members and 423 community controls) who met eligibility criteria of being at least 50 years old, had at least a high school education, and used English as their primary language were initially enrolled in 1984. A major re-recruitment effort in 1991 targeted subjects who had dropped out in the first years of the study. To attenuate self-selection bias and exercise effects due to the exercisers among the community controls, we also created groups of Ever-Runners and Never-Runners based on responses to the question at baseline: "Have you ever run for exercise for a period greater than one month?" The study was approved by the Stanford University Investigational Review Board, and each subject gave their informed consent.

### Data collection

Each subject completed annual, mailed health assessment questionnaires [12,13]. The questionnaire includes items on medical history, health status, exercise habits, history of musculoskeletal injuries, health care utilization, and demographic variables, such as height and weight, smoking, and alcohol use.

### Assessment of physical activity

Physical activity data were obtained from responses to the question: "How many minutes each week do you exercise vigorously (vigorous exercise will cause you to sweat, and your pulse, if taken, will be above 120). Include periods of rapid walking at work and in daily activities." Subjects indicated their participation in running, jogging, swimming, bicycling/stationary bike, aerobic dance/exercises, stair steppers, brisk walking, hiking/treadmill, racket sports, and other.

### Assessment of pain

Annually since 1987, pain was assessed using a visual analog scale (VAS) where 0 = no pain and 100 = worst pain. From 1987 through 1989, subjects responded to the question "How bad has pain or stiffness been in the past week?" and marked their response on the VAS. In 1987, the VAS anchors were: 0 = no pain or stiffness; and 100 = very severe pain or stiffness. In 1988 and 1989, the VAS anchors were: 0 = no pain; and 100 = severe pain. Beginning in 1990, a closed-end stem about the presence or absence of pain was added. If the patient affirmed they had pain, then they rated their amount of pain on the VAS. If they responded "No", then their pain score was assigned a value of zero.

### Statistical analysis

Differences between groups at first evaluation (baseline) were compared using chi-square and t-tests. Results are reported as mean (SE) or proportion. Longitudinal data were analyzed using generalized estimating equations (GEE) [14]. Separate analyses were conducted in which repeated measurements were coded by calendar year for questionnaire response and by age. Main-effect predictors were exercise group (Runners' Association/community controls), gender, baseline age, baseline body mass index (BMI; kg/m^2^), years of education, number of hospital days in the past year, and dichotomous variables for smoking, and history of arthritis, fractures, and cancer at baseline. Baseline values *y*_*t *= 1987 _were defined as weighted means [15],



For all analyses, exercise group and gender were combined to form a four-level classification factor. Two-way interactions of each predictor with this classification factor were also included. To reduce collinearity, each continuous predictor was dichotomized about its mean. This dichotomization also produced estimates of gender and exercise group main effects that were more meaningful.

Four analytic approaches were employed to help reduce the impact of possible self-selection bias. First, we conducted a separate analysis by Ever-Runner and Never-Runner classification as well as by Runners' Association and community controls groups (from original enrollment). This grouping expanded the cases to include individuals who self-selected to run at an earlier age and who stopped running because of pain or other reasons before entering the study. Second, we used covariate adjustment to account for baseline differences between groups. Direct standardization [16] was used to produce covariate-adjusted mean VAS pain by exercise group and by study year for statistically significant predictors that were identified by the above regression analyses. Data for the community controls group from the first year that VAS pain was observed (1987) served as the reference standard. In addition to providing adjustment for differences on covariates between Runners' Association and community controls groups, use of a standard taken at baseline also permitted adjustment for possible attrition bias. Third, we assessed attrition bias (because any differential attrition between groups could result in a secondary form of self-selection bias) by performing separate analyses limited to study subjects who completed all questionnaires in addition to all subjects (completers versus all). Finally, we used a longitudinal study design in which an adverse stimulus is expected to eventually result in a poor outcome regardless of initial self-selection bias if groups differ sufficiently in exposure to the stimulus. If running creates damage through accumulated trauma, then runners with about ten-fold the amount of exposure to such trauma should have increased pain over time, and any initial differences due to self-selection should narrow as the study progresses.

## Results

**Figure 1:**
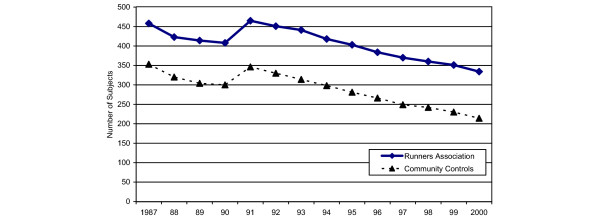
Sample size over time for Runners' Association members and community controls.

In 1987, the first year that VAS pain was assessed, 811 subjects returned questionnaires (458 Runners' Association members; 353 community controls). The 1991 re-recruitment efforts increased study enrollment to 881 subjects (496 Runners' Association members; 385 community controls). Fifteen subjects were excluded from these analyses because classification data for Ever-Runner versus Never-Runner were not available. Over the study duration, subject retention averaged more than 95% on an annual basis (and 98% of living subjects each year) as shown in Fig. 1. The mean (SE) years of follow up for Runners' Association and community controls were 11.4 (0.17) and 10.1 (0.22) (p < 0.05 for difference), respectively, and for Ever-Runners and Never-Runners it was 11.4 (0.16) and 10.5 (0.25) (p < 0.05 for difference), respectively. Data for this study are based on 866 subjects (492 Runners' Association and 374 community controls), who were also grouped as Ever-Runners (n = 565) and as Never-Runners (n = 301), for whom data were available.

Demographic characteristics at the beginning of the study are presented for the four study groups in Table 1. Overall, subjects were similarly well educated, but Runners' Association members and Ever-Runners were statistically younger, had lower BMI and baseline pain scores, ran more miles, exercised more minutes per week, and smoked less (all p < 0.05) relative to community controls and Never-Runners. History of arthritis was lower in Runners' Association members and Ever-Runners than community controls and Never-Runners, but statistically significant only for Ever-Runners versus Never-Runners. In quintiles of baseline exercise minutes/week, less than a fourth (22%, n = 37) of Ever-Runners and less than a tenth (7%, n = 12) of Runners' Association members were inactive, exercising less than 70 minutes a week (data not shown). In contrast, at the highest quintile, 88% (n = 155) of Runners' Association members and 91% (n = 159) of the Ever-Runners exercised between 355 and 2,119 minutes/week, indicating that subjects in both of these groups were very physically active. At the end of the study period, the groups had maintained similar levels of exercise minutes/week.

Baseline demographic characteristics for the two sets of groups by gender are shown in Table 2. For both Runners' Association members and Ever-Runners, the greater majority of subjects were male (approximately 83%), whereas in community controls and Never-Runners the sex ratios were more evenly split (56% and 50%, respectively). In females, Runners' Association members were younger, weighed less, reported less pain, fewer hospital days during the past year, ran more miles and exercised more minutes a week (all p < 0.05) than female community controls. Female Runners' Association members were also better educated, smoked less, and drank less alcohol than their community control counterparts (all p > 0.05) and there was a higher proportion of females with a history of arthritis and a lower proportion of females with a history of fractures in the community controls and Never-Runners compared to their counterparts, but these differences were statistically indistinguishable. Characteristics of male Runners' Association members versus community controls followed similar patterns, although differences in VAS pain and hospital days were not statistically significant, whereas education, smoking and alcohol were.

**Figure 2:**
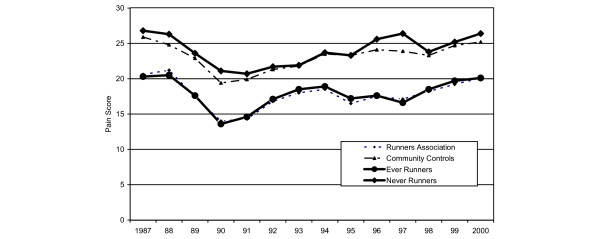
Adjusted mean visual analog scale pain scores over time by study group. For both comparison groups of runners (Runners' Association members and Ever-Runners), pain scores remained significantly lower over time (p < 0.01) when compared with community controls or Never-Runners.

Pain scores over time, adjusted for group, gender and baseline BMI, are presented in Fig. 2 for each study group (Runners' Association members, community controls, Ever-Runners, and Never-Runners). The statistically significant covariates, excluding time and group, were gender (p < 0.01), baseline BMI (p < 0.01), cigarette packs/day and number of hospital days (p = 0.02). For both comparison groups of runners (Runners' Association and Ever-Runners), pain scores remained significantly lower over time (p < 0.01) when compared with community controls or Never-Runners. The dip in scores between 1987 and 1991 is a result of the rephrasing and coding of the pain question as described earlier. Pain scores were consistently about 25% less in the exercising group throughout the period of observation.

**Figure 3:**
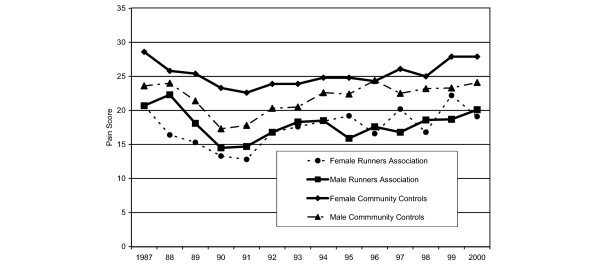
Adjusted mean visual analog scale pain scores over time by gender and study group. After adjusting for covariates, the community controls have more pain over time; however, female controls have the greatest self-reported pain, with female and male controls reporting significantly more pain than either female runners (p = 0.0048) or male runners (p = 0.004).

Because gender was a significant covariate, pain scores over time are presented by gender in Fig. 3, adjusted for covariates. Significant covariates, excluding time and group, are fracture in past year (p = 0.026) and the presence of arthritis (p < 0.001). As observed previously, community controls have more pain over time; however, female controls have the greatest self-reported pain, with female and male controls reporting significantly more pain than either female runners (p = 0.048) or male runners (p = 0.004). Similar results were observed for VAS pain scores by gender for Never-Runners and Ever-Runners (data not shown).

To evaluate the impact of study attrition and the possibility that withdrawal from the study might be associated with increased pain, we repeated the analyses by group and gender for study completers only. There were 61 female runners, 253 male runners, 84 female community controls and 116 male community controls who completed all questionnaires. In addition to time, group, and gender, presence of arthritis (p < 0.001) and education years (p = 0.036) were significant covariates. Similar to results of analyses using all available data, reduced levels of pain for male and female runners were observed in completers, although the only statistically significant difference over time is between female runners and male and female controls (p < 0.05).

**Figure 4:**
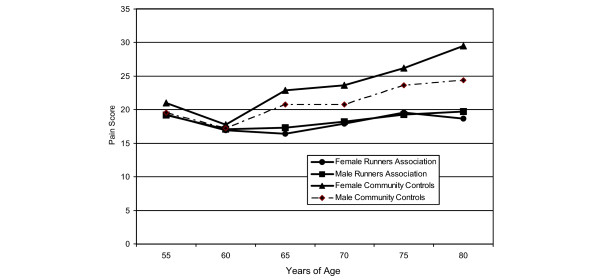
Adjusted mean visual analog scale pain scores by age, gender and study group. Significant increases in pain scores for female and male community controls and runners were found with increasing age, although rates of increase are relatively modest. Older female runners tend to have the greatest benefit, although these associations are statistically equivalent to differences in male and female runners and controls (p = 0.51).

Our final analyses tested the extent to which exercise and pain were affected by increasing age (Fig. 4). As in previous analyses, a history of arthritis and fractures were significant covariates (p < 0.001). There were significant increases in pain scores for female and male community controls and runners with increasing age, although the rates of increase are relatively modest. Older female runners tended to have the greatest beneficial impact, although these associations were statistically equivalent to differences in male runners and controls (p = 0.51).

## Discussion

This paper addresses the issue as to whether consistent vigorous exercise patterns over the long term are associated with greater or reduced musculoskeletal pain. In these cohorts, runners had substantially reduced pain levels compared with controls, which persisted over average ages of 62 to 76 years. Exercise was associated with a substantial and significant reduction in pain even after adjusting for gender, baseline BMI and attrition, and despite the fact that fractures, a significant predictor of pain, were slightly more common among runners. This relationship held as well when study completers only were evaluated.

Previous studies have indicated that men and women can differ in levels of self-reported pain and its importance [17-20]. In this study, male community controls reported less pain than their female counterparts. Female community controls and Never-Runners tended to report the highest levels of pain on average, whereas female runners appeared to receive the greatest benefit in reduced pain.

This study does not provide insight into the mechanisms that might underlie these results, although we have previously ruled out self-report bias between runners and non-runners by differential validation against spousal values [21]. A trend toward more frequent reports of a history of arthritis in controls could have played a role; fractures, however, were more common in runners and should have worked in the opposite direction. Other possible mechanisms include endorphin release, exercise protection against secondary fibromyalgia, increased resistance to musculoskeletal micro-injury, psychologically based increase in pain threshold, innately high pain threshold influencing decision to exercise vigorously, or other psychological mechanisms.

Musculoskeletal pain has also been shown to be associated with disability in older individuals and individuals with some chronic diseases [22,23]. But in a study of persons with rheumatoid arthritis, Ward and Leigh [22] noted that pain was a larger contributor to measurement of overall health status than physical disability and among both older male and female individuals. Leveille and colleagues [23] evaluated the presence of pain in women over 65 years of age and observed widespread musculoskeletal pain.

## Conclusion

The primary finding from this investigation is that while pain does increase with age in subjects in all study groups, there was no progressive increase in musculoskeletal pain in older adults who participated in regular vigorous exercise, including running, compared with those who did not. Initial differences favoring exercisers were shown to be maintained over time. As pain and disability are linked, our findings add to the evidence that morbidity associated with aging can be reduced by participating in regular aerobic activity.

## Abbreviations

BMI = body mass index; GEE = generalized estimating equations; VAS = visual analog scale.

## Competing interests

The authors declare that they have no competing interests.

## Authors' contributions

BB performed statistical analyses, interpretation of data, and drafting of the manuscript; JFF participated in study design, interpretation of data, and drafting of the manuscript; DL participated in study design, statistical analyses, interpretation of data, and drafting of the manuscript. All authors have read and approved the final manuscript.

## Figures and Tables

**Table 1:** Baseline characteristics of study groups

	Runners' Association (n = 492)	Community controls (n = 374)	Runners	
			Ever (n = 565)	Never (n = 301)

Male (%)	83	56	84	50
Mean (SE)				
Age (years)	61.6 (0.25)^a^	65.1 (0.36)	61.7 (0.25)^a^	65.6 (0.41)
Education (years)	16.6 (0.11)	16.7 (0.13)	16.7 (0.10)	16.5 (0.15)
BMI (kg/m^2^)	22.9 (0.11)^a^	24.2 (0.18)	23.0 (0.11)^a^	24.3 (0.21)
Pain (VAS 0–100; 0 = no pain)	20.7 (1.05)^a^	25.7 (1.27)	20.4 (0.98)^a^	26.6 (1.44)
Hospital days/past year	0.27 (0.05)	0.34 (0.08)	0.24 (0.05)	0.41(0.09)
Running miles (week)	25.5 (0.65)^a^	2.1 (0.30)	23.1 (0.64)^a^	1.06 (0.29)
Exercise minutes (week)	313.6 (9.2)^a^	123.2 (6.8)	290.8 (8.7)^a^	119.9 (7.2)
Cigarette (packs/day)	0.02 (0.01)^a^	0.08 (0.02)	0.01 (0.01)^a^	0.11 (0.02)
Alcohol (oz./day)	1.08 (0.06)	1.22 (0.06)	1.11 (0.05)	1.22 (0.07)
Subjects (%) with history of				
Arthritis	35	41	35*	43
Fractures	53	47	52	48
Cancer	0.002	0.005	0.004	0.003

^a^p < 0.05 Runners' Association versus community controls or Ever-Runners versus Never-Runners. BMI, body mass index; VAS, visual analog scale.

**Table 2:** Baseline characteristics by study group and gender

	Runners' Association		Community Controls		Ever-Runners		Never-Runners	
	Female (n = 82)	Male (n = 410)	Female (n = 163)	Male (n = 211)	Female (n = 93)	Male (n = 472)	Female (n = 152)	Male (n = 149)

Mean (SE)								
Age (years)	59.9 (0.5)^a^	62.0 (0.3)^a^	65.5 (0.5)	64.6 (0.5)	60.3 (0.5)^a^	62.0 (0.3)^a^	65.7 (0.6)	65.5 (0.6)
Education (years)	16.3 (0.3)	16.6 (0.1)^a^	15.8 (0.2)	17.5 (0.2)	16.4 (0.3)^a^	16.8 (0.1)	15.7 (0.2)	17.3 (0.2)
BMI (kg/m^2^)	21.6 (0.2)^a^	23.2 (0.1)^a^	23.2 (0.3)	25.0 (0.2)	21.6 (0.2)^a^	23.3 (0.1)^a^	23.4 (0.3)	25.3 (0.3)
Pain (VAS 0–100; 0 = no pain)	20.9 (2.6)^a^	20.7 (1.2)	27.6 (2.1)	23.0 (1.6)	19.6 (2.4)^a^	20.5 (1.1)	28.8 (2.1)	24.3 (1.9)
Hospital days/past year	0.09 (0.1)^a^	0.31 (0.1)	0.45 (0.1)	0.25 (0.1)	0.08 (0.0)^a^	0.28 (0.1)	0.48 (0.2)	0.33 (0.1)
Running miles (week)	19.4 (1.3)^a^	26.9 (0.7)^a^	0.99 (0.4)	3.0 (0.4)	17.4 (1.34)^a^	24.3 (0.7)^a^	0.9 (0.4)	1.3 (0.4)
Exercise minutes (week)	310.8 (19.8)^a^	314.2 (10.3)^a^	115.8 (10.0)	129.0 (9.1)	283.9 (18.9)^a^	292.2 (9.7)^a^	118.2 (10.8)	121.6 (9.4)
Cigarette (packs/day)	0.09 (0.04)	0.01 (0.04)^a^	0.13 (0.0)	0.05 (0.0)	0.05 (0.0)^a^	0.01 (0.0)^a^	0.16 (0.0)	0.06 (0.0)
Alcohol (oz./day)	1.0 (0.1)	1.1 (0.1)^a^	1.0 (0.8)	1.4 (0.1)	0.98 (0.1)	1.14 (0.1)^a^	1.01 (0.1)	1.46 (0.1)
Subjects (%) with history of								
Arthritis	45	33	47	36	42	33	49	36
Fractures	54	53	41	52	51	52	42	54
Cancer	0	0.002	0	0.009	0	0.004	0	0.007

^a^p < 0.05 male or female Runners' Association versus matched community controls or male or female Ever-Runners versus matched gender Never-Runners. BMI, body mass index; VAS, visual analog scale.
